# Rational design for multifunctional non-liposomal lipid-based nanocarriers for cancer management: theory to practice

**DOI:** 10.1186/1477-3155-11-S1-S6

**Published:** 2013-12-10

**Authors:** Sabrina Valetti, Simona Mura, Barbara Stella, Patrick Couvreur

**Affiliations:** 1 Univ Paris-Sud, Faculté de Pharmacie, 5 rue Jean-Baptiste Clément, 92296 Châtenay-Malabry cedex, France; 2 CNRS UMR 8612, Institut Galien Paris-Sud, 5 rue Jean-Baptiste Clément, 92296 Châtenay-Malabry cedex, France; 3 Dipartimento di Scienza e Tecnologia del Farmaco, Università di Torino, via P. Giuria 9, 10125 Torino, Italy

**Keywords:** lipid, nanocarrier, passive or active targeting, diagnosis, theranostic

## Abstract

Nanomedicines have gained more and more attention in cancer therapy thanks to their ability to enhance the tumour accumulation and the intracellular uptake of drugs while reducing their inactivation and toxicity. In parallel, nanocarriers have been successfully employed as diagnostic tools increasing imaging resolution holding great promises both in preclinical research and in clinical settings. Lipid-based nanocarriers are a class of biocompatible and biodegradable vehicles that provide advanced delivery of therapeutic and imaging agents, improving pharmacokinetic profile and safety. One of most promising engineering challenges is the design of innovative and versatile multifunctional targeted nanotechnologies for cancer treatment and diagnosis. This review aims to highlight rational approaches to design multifunctional non liposomal lipid-based nanocarriers providing an update of literature in this field.

## Introduction

Cancer is the first leading cause of death in developed countries and the second one in developing countries, accounting for 7.6 million deaths (around 13% of all deaths) in 2008[1]. The World Health Organization predicts that by 2030 12 million of all deaths worldwide will be due to cancer [1]. Far from being a "modern" disease, cancer is one of the oldest maladies even if it start receiving more and more attention only when other severe killer diseases (such as tuberculosis, dropsy, cholera, smallpox, leprosy or pneumonia) had been eradicated. Despite an old and impatient battle, in which the international scientific and non-scientific committees are engaged, the knowledge of cancer's biology and the discovery of new molecules are unlikely to fully eradicate it. Even if new molecules are discovered to treat cancer, the efficacy of conventional chemotherapeutics is hampered by the following limitations: i) drug resistance at the tumour level due to physiological barriers (*i.e.;*non-cellular based mechanisms) ii) drug resistance at the cellular level (i.e.cellular mechanisms) and iii) non-specific distribution, biotransformation and rapid clearance of anticancer drugs in the body [2]. The process that drives a drug to the target is indeed dependent on drug physico-chemical properties that affect its stability in the systemic circulation, the extravasation and the intratumoral distribution, also leading to undesired side effects [2]. To overcome these limits, the "magic bullet" theory, which refers to a drug which goes straight to its specific target, was postulated at the beginning of the XXth century [3]. In the past decades the application of this concept has led to the development of a plethora of colloidal systems aimed at deliver the drug exclusively to the diseased tissues, thus reducing systemic toxicity. In particular, in the past 35 years, cutting-edge research based on multidisciplinary approaches has been led to the development of nanoscaled drug carriers for medical application [2,4]. The first paper on nanoparticles was published in 1976 by Peter Speiser, a pioneer in the concept of nanoparticles: it focused on the development of nanoparticles for vaccination purposes, aiming at a slow release profile of the antigen thus leading to a better immune response [5]. Later, Couvreur et al [6] discovered the lysosomotropic effect of nanoparticles and for the first time published that nanocapsules were able to introduce compounds into cells which do not spontaneously accumulate intracellularly.

Rapidly, nanoparticles (NPs) found important application in cancer therapy due to numerous advantages that they offer over the free drugs [Table 1][7-10]. Some engineered nanocarriers were been approved by the FDA (Doxil^® ^[11], Daunoxome^® ^[12], Abraxane^® ^[13], Genexol^® ^[14], Marqibo^® ^[15]). Marqibo^® ^is a vincristine loaded liposomal formulation made of sphingomyelin and cholesterol approved in 2012 for the treatment of adult patients with Philadelphia chromosome-negative (Ph -) acute lymphoblastic leukaemia[15].

**Table 1:** Nanocarriers advantages and properties required for clinical translation [22]

Advantages offered by nanocarriers

• Prevention of undesired drug interaction with the biological environment (*i.e.*, drug inactivation by metabolization) • Control on pharmacokinetic/pharmacodynamic parameters • Enhanced drug accumulation at the tumor target site and improved intracellular uptake • Safety (*i.e.; *decrease of drug toxicity and side-effects).

Aside from therapeutic use, in recent years nanocarriers have also been employed as imaging tools which hold great promises both in preclinical research and in clinical settings[16-21]. Nanoparticles for diagnostic purposes have now been marketed for 10 years[4]. The encapsulation of different imaging contrast agents (e.g., paramagnetic metal ions, superparamagnetic iron oxide nanoparticles (SPIOs), Near Infra-Red (NIR) probes, radionuclides) in nanocarriers makes possible to enhance the signal to noise ratio in the targeted tissue compared to the surrounding health one. The increase of imaging resolution highlights small lesions which are undetectable with traditional methods.

At the moment, biodegradable polymers or lipid-based colloids are the only drug vehicles approved for clinical use. These materials offer promising possibilities to assure specific drug accumulation at the tumour site, improving the pharmacokinetic profile and safety of both drug and contrast imaging agents[22].

The present review is focused on lipid-based nanocarriers which have classically received great attention due to their biodegradability, biocompatibility and targetability[23]. Lipid nanocarriers used for drug delivery purposes include liposomes, micelles, nanoemulsions, nanosuspensions, solid-lipid nanoparticles and lipoproteins-containing systems. Liposomal systems attract a great deal of interest and a simple research on the PubMed database reveals that more than 150 review articles have been published within this field in the last year alone. Consequently, we decided to limit the present review to non-liposomal lipid-based nanocarriers. After a short description of these drug nanocarriers, their applications as multifunctional tools for therapeutic and/or diagnostic applications in cancer management are reviewed.

### Non-liposomal lipid-based nanocarriers

**Figure 1:**
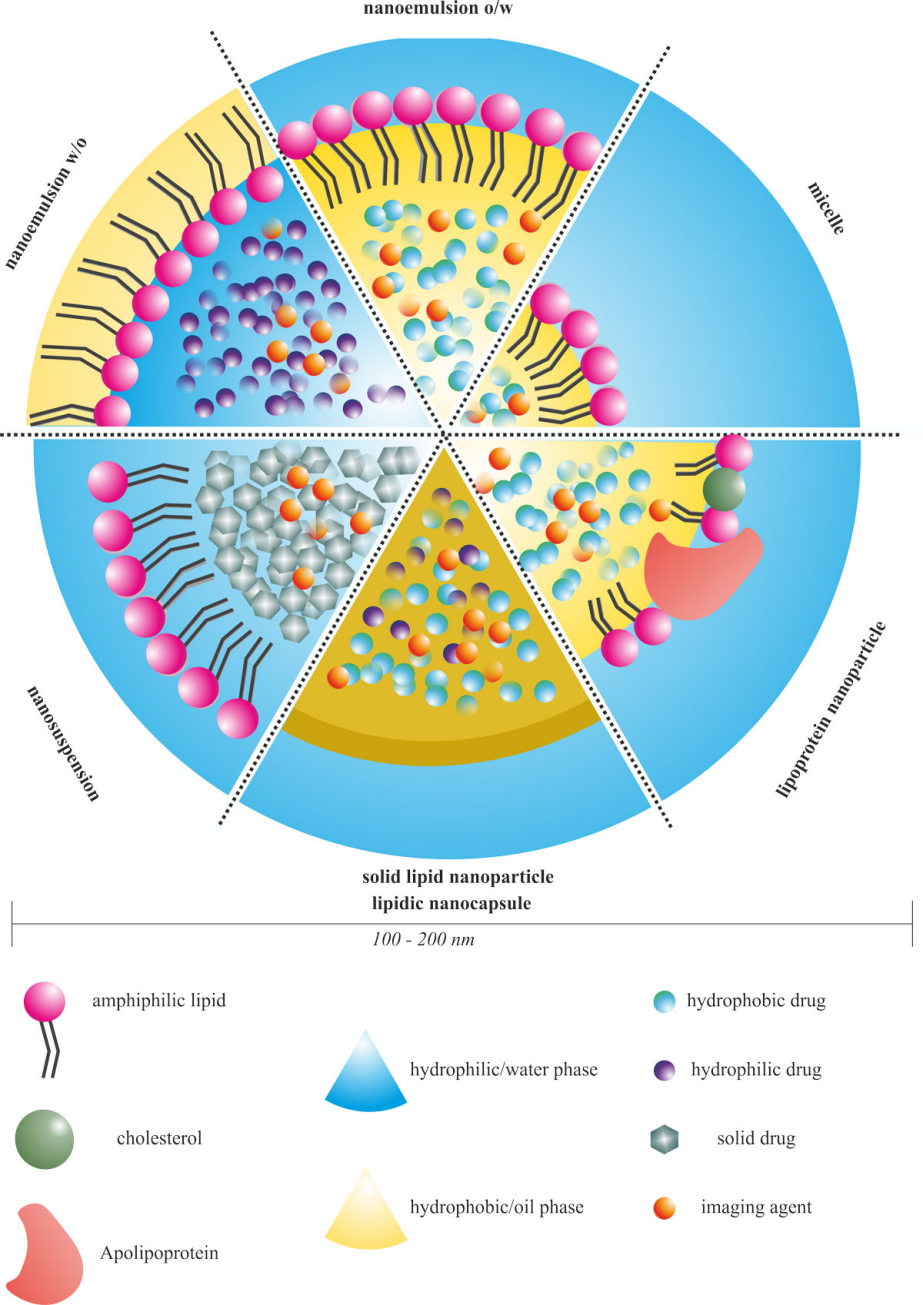
**Schematic representation of non-liposomal lipid-based nanocarriers**.

A broad range of lipid nanocarriers is currently used for drug delivery purposes. Although sometimes the boundaries between categories are not clearly defined, they can be classified into micelles, nanoemulsions, nanosuspensions, solid lipid nanoparticles, lipid nanocapsulesand lipoproteins (Figure 1).

Micelles are colloidal dispersions, which form spontaneously from amphiphilic or surfactant agents at certain concentrations and temperatures. They are characterized by two distinct portions with opposite affinities towards a given solvent. Lipid micelles are formulated adding phospholipids or long-chain fatty acids in the presence of appropriate surfactants[24]. At low concentrations, in an aqueous medium amphiphilic molecules exist separately and aggregation takes place within concentrations above to critical micelle concentration[25].

Micelles possess a hydrophobic core and hydrophilic shell; they have been successfully used as pharmaceutical carriers for water-insoluble drugs or molecular imaging probes[26]. Thanks to their small size (from 5 to 100 nm) they demonstrated a very efficient and spontaneous accumulation in pathological areas with compromised vasculature. However, due to the limited size of their core they cannot load high amount of drugs. Lipid micelles are formulated adding phospholipids The formation of micelles is driven by the decrease of free energy in the system because of the removal of hydrophobic fragments from the aqueous environment and the reestablishment of a hydrogen bond network in water. Lipid-based micelle preparation is a simple process, often base on a detergent or water-miscible solvent removal method that gives spontaneous formation of colloids with very similar diameters in aqueous media.

Nanoemulsions are transparent or translucent oil-in-water (o/w) or water-in-oil (w/o) droplets that can encapsulate either lipophilic and hydrophilic drugs or imaging agents in the oil or in the aqueous phase, respectively [27-29]. They are formulated from lipid components through high-energy methods (e.g., high-pressure homogenization, microfluidization or ultrasonification in order to obtain small size droplets) or through low-energy methods (e.g., spontaneous emulsification, solvent-diffusion method and phase-inversion temperature for labile drugs)[30]. Advantages of nanoemulsions over macroemulsions include higher surface areas and free energy without the inherent creaming, flocculation, coalescence and/or sedimentation[27].

Likewise, nanosuspensions are sub-micron colloidal dispersions of particles of drug stabilized by surfactants (e.g., soya lecithin, mainly composed phospholipids). High pressure and multiple high-energy passes are often required for their production, owing to the drug crystal binding and its stabilization in the colloidal system[31]. Even if they could be prepared directly by crystallization or precipitation, high pressure homogenization is the most frequently employed in large-scale production[32]. They are usually used as injectable dosage forms for poorly soluble drugs. In the case of high melting point compounds, the nanosuspensions allow preserving the crystalline state to obtain the small size required for an intravenous administration. Taking advantage of the absence of any solvent, the nanosuspensions possess higher drug loading compared to nanoemulsions[27].

Solid lipid nanoparticles (SLN) can be considered as nanosuspensions with a solid lipid core stabilized by surfactants[33]. They are typically formed by heating an aqueous lipid mixture above the melting point of the lipid, adding drug, homogenizing and finally cooling to freeze the drug within the solid lipid spheres. Other procedures like microemulsification, high-pressure homogenization, solvent emulsification-evaporation and "coacervation" method have been proposed for the preparation of SLN[34-36]. A broad range of biocompatible and biodegradable lipids that remains in solid form at physiological temperatures has been used for SLN: fatty acids (e.g., stearic acid, palmitic acid), triglycerides (e.g., trilaurin, tripalmitin, and tristearin) and satured fatty acids (e.g., glycerol behenate, and cetylpalmitate).

SLN show a significant versatility for drug or contrast agent delivery since they can load lipophilic, hydrophilic, amphiphilic as well as charged molecules. They are characterized by an important physical stability that offers several technological advantages, including (i) better storage stability in comparison to liposomes, (ii) easy management in large-scale production and (iii) possibility of lyophilization [37,38]. Numerous investigations have demonstrated that SLN can very efficiently control drug release, also improving drug accumulation into the tumour, along with a concomitant minimization of severe side effects and low toxicity of the carrier[39,40]. Despite these advantages, the solid crystalline core of SLN can present several drawbacks, such as problems of reproducibility in the particle growth, possibility of polymorphic transitions, which can induce drug expulsion during storage, and low drug incorporation capacities[41].

Lipid nanocapsules (LNC) are constituted by an oily core surrounded by a tensioactive-based rigid membrane which represents a hybrid structure between polymeric nanocapsules and liposomes[42]. Empty or drug-loaded LNC, with a diameter below 100 nm and a narrow size distribution, can be prepared by a phase inversion temperature process and show long physical stability (> 18 months)[42]. Different anticancer drugs, [43-45] nucleic acids [46] or, imaging agents [47-49] have been encapsulated in the lipid core of these nanoformulations. Surface modification with PEG chains has been also described[44]. Promising results have been obtained both *in vitro *on several cell lines and on *in vivo *models of experimental cancers.

Natural lipoproteins present in the blood as macromolecular carriers for hydrophobic lipids have also been employed as nanocarriers. Lipoproteins are classified in four categories depending on the density, from the largest diameters and lowest density: chylomicrons, very low-density lipoprotein (VLDL), low-density lipoprotein (LDL) and high density lipoprotein (HDL). They are basically formed by a core of triacylglycerides and cholesterol esters coated by a phospholipid and apolipoprotein shell [50]. By mimicking the endogenous shape and structure of lipoproteins, lipoprotein-inspirated nanocarriers could escape mononuclear phagocyte system recognition, thus remaining in the blood stream for an extended period of time, [51] ranging from 10 to 12 h in rodents [52] and up to 5 days in humans, as demonstrated by a clinical study in which autologous biotinyl-HDL3 was injected to five normolipidemic male volunteers as a probe for the determination of nanocarrier turnover [52,53]. LDL and HDL, mostly used for their diameters lower than 40 nm, can be loaded with drugs or imaging agents through covalent linkage with the phospholipid or protein material, intercalation of the molecules into the phospholipid shell or encapsulation in the nanoparticle core.

This approach provides a highly versatile natural nanoplatform for the delivery of poorly soluble drugs or imaging agents [54]; however, one potential hurdle in developing lipoproteins as clinically viable nanocarriers lies in the fact that lipoproteins are isolated from fresh-donor plasma, which might result in batch-to-batch variations thus posing several scale-up challenges[55].

### Targeted non-liposomal lipid-based nanocarriers

Nanoscaled systems appear as an attractive approach to overcome the limitations associated to conventional drug delivery strategies. However, the existence of endogenous self-defence mechanisms able to recognize either viral/bacterial or synthetic exogenous particles may hinder their effectiveness or cause side undesirable effects. The mononuclear phagocyte system is a part of the immune system that consists of the phagocytic cells (monocytes, macrophages and Kupffer cells) widely distributed and strategically placed in many tissues of the body (lymph nodes, spleen and liver) to recognize and neutralize foreign particles[56]. The recognition by these cells is promoted by the adsorption of specific proteins (renames "opsonins"), capable of interaction with specific plasma membrane receptors on monocytes and various subsets of tissue macrophages[57-59].

In the case of infectious diseases, this mechanism provides an opportunity for the efficient delivery of therapeutic agents to these cells by using colloidal drug delivery systems[60,61]. However, in cancer treatment, the rapid sequestration of intravenously injected colloidal particles by liver and spleen decreases drug accumulation at the tumour site. Thus, the engineering of colloidal carrier systems which avoid rapid recognition by Kupffer cells and show long blood circulation time (i.e., Stealth^® ^nanoparticles) is essential[62]. To this aim, several approaches have been investigated to modify the surface properties of the nanocarriers by using emulsifying agents or copolymer nonionic surfactants such as poloxamers and poloxamines, in order to block the opsonization process[63,64]. One of the most successful methods is the anchoring onto the nanoparticle surface, of a hydrophilic and flexible polymer, like polyethylene glycol (PEG) or its derivatives[65-71].

**Figure 2:**
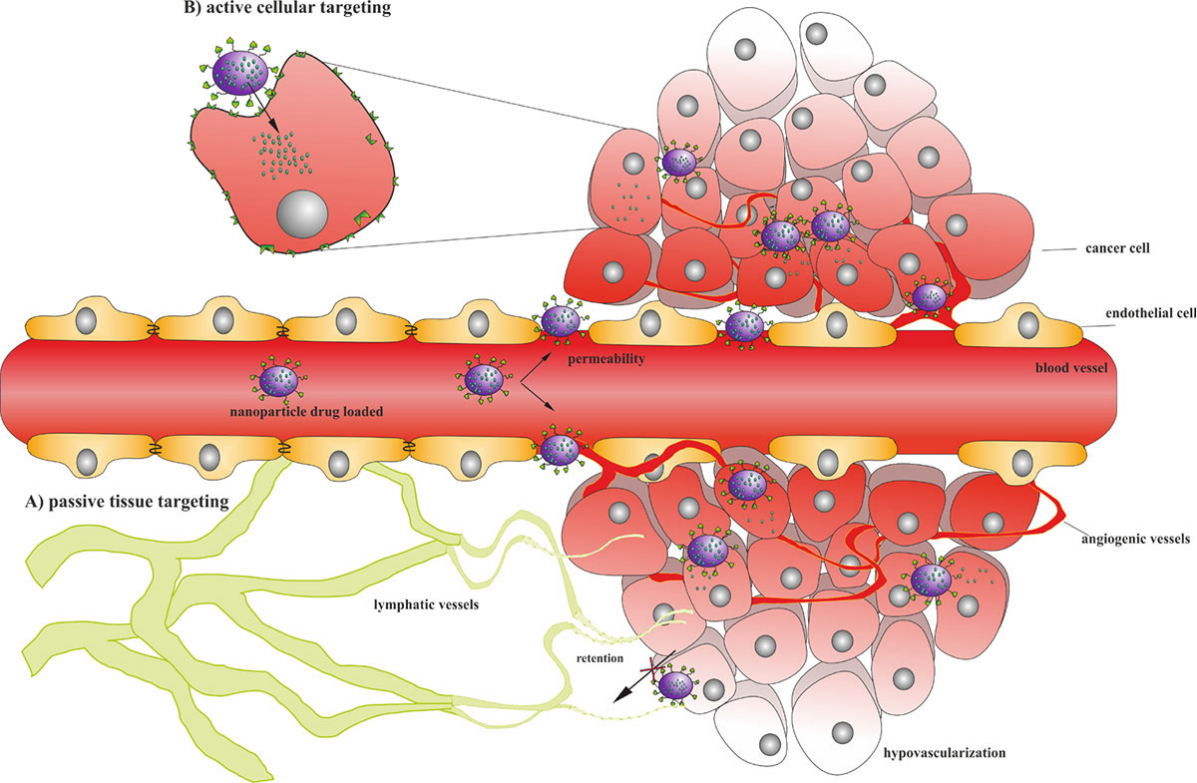
**(A) Passive targeting**. Healthy blood vessels are regular and continuous with tight endothelial junctions between cells. Conversely, the angiogenic vessels show gross architectural changes, such as large intracellular pore, presence of interrupted endothelium and incomplete basement membrane, allowing the extravasation of nanoparticles from blood vessels. In the tumour stroma nanoparticles remain trapped due to higher interstitial pressure, due to a lack of effective lymphatic drainage coupled with lower intravascular pressure. These pathophysiological characteristics enhance the tumour site accumulation of nanoparticles. However, aside to the well perfused and rapidly growing regions, a non-uniform tissue oxygenation due to the vascular heterogeneity led to the presence of poorly perfused, often necrotic areas in which the efficacy of the treatment is hampered. **(B) Active targeting**. In order to improve the intracellular delivery of the drug, nanoparticles could be functionalized with specific ligands that specifically bind receptors expressed primarily on malignant cells leading to receptor-mediated internalization, which is often necessary to release drugs inside the cells.

Surface modification of nanoparticles not only confer significantly reduced mononuclear phagocyte system uptake, better stability and enhanced circulation time, but also result in an increased accumulation of the stealth particles in the tumour[72,73]. Compared to healthy tissues, tumours show high irregular vessels with abnormal heterogeneous density, large pores on the endothelial walls, reduced lymphatic drainage and higher interstitial pressure[74]. Due to this enhanced permeability and retention (EPR) effect, drug-loaded nanocarriers are able to accumulate at the tumour site by passive targeting (Figure 2A)[75,76].

However, PEGylation presents some important limits and drawbacks concerning the translation to the clinic. The observed discrepancy between preclinical and clinical results could, indeed, be attributed to the different progression rate of tumours models in animals and those of human patients, an important factor for EPR based anticancer nanomedicines[77]. In addition, it is already well known that the Doxil^®^, a PEGylated doxorubicin liposomal formulation, is able to trigger complement activation in human serum, leading to a pseudoallergic reactions called "complement activation-related pseudoallergy" (CARPA) which is associated with cardiopulmonary disturbance and other related symptoms of anaphylaxis[78]. For instance, a recent study showed that the extent of complement activation was correlated to the amount of methoxyPEG 2000 (or 5000) at the surface of the lipid carrier[79]. Moreover, relying only on the EPR effect and therefore on tumour anatomy, in some cases passive targeting did not allow therapeutic drug amounts to reach the target site. Indeed, the physiology of tumours and especially fibrosis, hypovascularization [80,81] and the presence of extracellular matrix, [77] a highly interconnected network of collagen fibers, obstruct the nanoparticles to reach cancer cells. That seems to be the cause of the failure in pancreatic adenocarcinoma treatment[82,83].

One of the major requirements for a successful cancer therapy is its ability to selectively kill cancer cells with minimal damage to healthy tissues[84]. In cancer cells, the extracellular leaflet of the plasma membrane is not characterized by unique molecular target but rather by overexpressed antigens that are relatively down regulated in healthy cells[85,86]. Thus, functionalization of nanocarrier surface with various targeting moieties that specifically bind the receptors mainly expressed on malignant cells has been widely investigated as valuable strategy to achieve an active targeting to cancer cells (Figure 2B)[87,88]. Receptor-mediated internalization of nanocarriers would then allow efficient drug release inside the cell. Several ligands that belong to the families of small molecules, polysaccharides, peptides, proteins or even antibodies have been used for targeted nanocarriers. A broad range of techniques could be employed to investigate specific homing devices such as (i) antibody-based screens [89], (ii) cloning strategies [90], (iii) in vivo biotinylation and (iv) phage-displayed peptide libraries[91]. In literature, several synthesis methods and coupling strategies are described to achieve the desired macromolecular architecture and display the homing device on the surface of nanocarriers (for a systematic review see [88]). Furthermore, nanoparticles generally carry more than a single targeting ligand molecule thus allowing multivalent binding, which improves targeting efficacy with high binding constants[92].

Lipid-based nanocarriers have been successfully employed for active targeting. The first example was published in Science in 2002[93]. In this study, cationic NPs were prepared by self-assembly and polymerization of appropriate lipid molecules and then functionalized by conjugation of a trivalent lipid with the integrin ανβ3 ligand for endothelial cell targeting (ανβ3-NPs). The expression of ανβ3 integrins in 25% of human tumours (e.g., melanoma, glioblastoma, ovarian, breast cancer) makes them a successful choice for the design of targeted drug delivery systems[94,95]. These actively targeted SLNs enabled selective gene delivery both in vitro (towards ανβ3-expressing M21 human melanoma cells) and in vivo (towards angiogenic blood vessels in mice bearing ανβ3-negative M21-L melanomas). The therapeutic efficacy was then tested injecting NPs conjugated with the mutant Raf-1 gene (ανβ3-NPs/ Raf (-)) that blocks endothelial signalling and angiogenesis. Ανβ3-NPs/ Raf (-) decreased angiogenesis, leading to tumour cell apoptosis and sustained regression of established primary and metastatic tumours. In a competitive assay experiment, treatment with 20-fold molar excess of soluble targeting ligand led to a tumour burden similar to that observed in control mice, demonstrating that the efficacy of targeted NPs resulted from ανβ3 specific recognition[93]. The well-known peptide sequence RGD (Arg-Gly-Asp), which recognizes the ανβ3 integrins, was identified 20 years ago[96]. Its cyclic form (cRGD), designed from the peptides developed by Kessler's group, provides easy conjugation to imaging and/or therapeutic moieties[41,94]. The importance of the peptide sequence for specific receptor targeting was demonstrated using negative control peptides which differ from the positive one only in few amino acids [41]. Lipid NPs were functionalized with both cRGD targeting ligand (SLN-cRGD) and cRAD peptide negative control (SLN-cRAD)[41]. Results in vitro on HEK293(β3) cells line (human embryonic kidney genetically modified which strongly express ανβ3 integrins) showed specific targeting of SLN-cRGD in comparison with SLN-cRAD and non-functionalized SLN after incubation with cells at 4°C or 37°C. Nanoparticle internalization was inhibited by pre-saturation of cells with free cRGD, demonstrating the key role of ανβ3 integrins. Similarly, an active accumulation of cRGD-targeted particles was observed in HEK293(β3) xenografts-bearing mice after intravenous injection[41]. However, cRGD targeting failed in a murine mammary carcinoma model clearly demonstrating that cancer physiopathology is a crucial parameter for cRGD targeting efficacy[41,97]. Efficient drug targeting requires the increased accumulation of the drug at the tumour site thanks to the EPR effect, followed by a facilitated cellular uptake through ligand-mediated endocytosis[22,98]. The proof of concept has been provided by Wang et al. [98] using passively and actively targeted lipid-based-nanosuspensions (LNSs) loaded with docetaxel. LNS modification with PEG moieties conferred stealth properties while further conjugation of PEG chains with folic acid enabled to achieve active targeting properties. Folic acid (FA) is widely used as targeting ligand due to the overexpression of FA receptors (FR) in several human cancer cells, including malignancies of the ovary, brain, kidney, breast, lung and myeloid cells[99]. FR binding affinity (Kd = 1 × 1−10 M) does not appear to be affected by conjugation to the nanocarriers, [98,100]. FA-functionalized systems represent an effective strategy for specific delivery of therapeutic agents to tumours[98,101-105]. Therefore, compared to non-functionalized LNS, targeted LNS showed a slightly higher toxicity on mouse melanoma B16 cells overexpressing FR, which was probably due to a synergy between the passive and active targeting[98].

FA was also used to decorate lipid-based nanoparticles made of (DSPC)/triolein/ cholesterol oleate/polyethylene glycol cholesterol (PEG-Chol) (40:40:18:2.5, mole: mole), in which a paclitaxel prodrug, the paclitaxel-7-carbonyl-cholesterol (Tax-Chol), was encapsulated in the lipid phase. In FA targeted formulations, 20% of the PEG-Chol was replaced by folate-PEG-Chol (f-PEG-Chol)[106]. The incorporation of a lipid paclitaxel prodrug was chosen as strategy to overcome the paclitaxel propensity to precipitate and increase formulation stability. The FR-targeted LNPs showed enhanced activity against FR (+) tumour-bearing mice also inducing an effective extension of their survival[106]. FA has been also conjugated to the Lys residues of the apolipoprotein B (apoB-100) to develop an actively targeted LDL-based nanoplatform[107]. LDLs possess an intrinsic tumour targeting property due to the overexpression of the LDL receptor (LDLr) in various tumour cells, which was attributed to the large amount of cholesterol and fatty acids required for sustaining the rapid tumour proliferation[108]. Although this approach might provide a targeted delivery of drugs and diagnostic agents to tumours, the application of LDL-like NPs is clearly limited to the dysregulation of the LDLR associated with several diseases [107]. Concerning FA-LDL, internalization studies performed on FR-overexpressing and FR-nonexpressing cells confirmed that the FA-targeted LDL-like NPs uptake was driven by the FR receptor[107].

EGFR is a transmembrane tyrosine kinase receptor overexpressed in a wide range of cancers including breast, ovarian, bladder, head and neck, glioma, pancreatic, kidney, lung and prostate, making it an attractive target for both therapeutic and diagnostic applications [109-111]. An example of EGFR-directed nanocarriers was provided through the functionalization of doxorubicin or carmustine-loaded cationic SLN with specific monoclonal antibody against EGFR for the treatment of brain glioblastoma multiforme[112,113]. Exposure to targeted nanoparticles resulted in higher inhibition of U87MG human glioblastoma-astrocytoma cells compared to non-targeted control NPs. Although these nanoparticles improved the administration of hydrophobic drugs, such as carmustine, allowing intravenously injection, an in vivo proof of evidence of the increased accumulation at the tumour site has not been provided yet. More recently, a new class of proteins known as affibody molecules has been introduced as an alternative approach to antibodies for EGFR-targeted systems [114,115]. These affibodies are composed of 58 amino acid residues bundled in a three-helix scaffold, a structure derived from the staphylococcal protein A Z-domain, which is an engineered variant of a gene encoding five highly homologous Ig-binding domains [116]. Taking inspiration from the advances in protein library technology, the Z domain was employed to design a novel class of high-affinity molecules. For example, the EGFR-binding Z domain was employed as homing device for the delivery of therapeutic agents towards a wide range of EGFR-overexpressing cancer cells [84]. To further improve binding efficiency, a heptameric EGFR-binding ligand was developed by fusing a heptamerization domain with an EGFR-binding Z domain. This heptameric EGFR-binding targeting ligand was used to decorate the surface of nickel-loaded lipid-based oil-filled nanoparticles (Ni-LNPs) [84]. Nanoparticles were prepared from warm oil/water (o/w) microemulsion technique using polyoxyethylene (20) stearyl ether, D-alpha-tocopheryl polyethylene glycol 1000 succinate (TPGS) and a mixture of caprylic and capric fatty acid triglycerides. In vitro cell uptake studies showed up to 90% internalization of the EGFR-targeted Ni-LNPs into the EGFR overexpressing A431 human epidermoid carcinoma cells, while a significantly lower uptake (10%) was observed with untargeted Ni-LNPs. The targeting efficiency of the novel heptameric Z-EGFR domain was also demonstrated in vivo with an almost two-fold increase of intracellular Ni accumulation in tumour cells[84].

The CD44 receptor-hyaluronic acid (HA) interaction has also been investigated for cancer targeting[117]. HA is an anionic, non-sulphated glycosaminoglycan distributed throughout connective, epithelial and neural tissues[118]. Contrary to HA oligomer, the native high molecular weight HA is a "bioinert" component that does not induce inflammation, proliferation or proangiogenic effect[119,120]. HA has been used as homing device able to target CD44-expressing tumour initiating cells[121]. Moreover, due to its hydrophilicity it could prevent opsonin adsorption by steric repulsion, allowing to reduce mononuclear phagocyte system uptake[122]. Thus, HA-Ceramide-based self-assembled NPs loaded with docetaxel [118] and doxorubicin[123]was developed. Ceramides, which are composed of sphingosine and fatty acid triglycerides, are cellular membranes component which play a role as cellular signalling molecules involved in the regulation of differentiation, proliferation and programmed cell death[124]. In vitro studies on several cells lines showed that the cellular uptake of docetaxel and doxorubicin-loaded HA-ceramide nanoparticles was driven by CD44 receptor-mediated endocytosis[118,123]. The in vivo tumour targetability for the docetaxel-loaded nanoparticles labelled with a NIR fluorescence die (cyanine 5.5) showed interaction between HA and CD44 receptors in MCF-7/ADR tumour bearing mice. Doxorubicin-loaded HA-ceramide nanoparticles showing PEG chains at their surface demonstrated an increased therapeutic efficacy in tumour-bearing mice, probably due to the improved half-life and reduced clearance of doxorubicin together with its tumour accumulation by passive and active targeting[123].

Galactose and galactosamine are also interesting ligands to target cancer cells which overexpress the asialoglycoprotein receptor (e.g., hepatic and cervical cancer cells)[88,125]. For efficient hepatocyte targeting, galactose was linked to the distal end of the PEG chains at the surface of DOTAP/DOPE lipid nanocapsules encapsulating DNA[126]. In primary hepatocytes, such functionalized lipid nanocapsules were found to increase by 18-fold the luciferase expression compared to non-galactosylated ones[126].

Galactoside functionalization of SLN loaded with taspine, a bioactive aporphine alkaloid that inhibits tumour angiogenesis and controls tumour growth, [127] enabled a 3-4-fold increase of drug accumulation in the liver of healthy mice[128]. Docetaxel-loaded SLN were instead targeted to hepatic cells using the galactosylated dioleoylphosphatidyl ethanolamine (DCT-tSLN)[129]. DCT-tSLN showed higher cytotoxicity on hepatocellular carcinoma cell line BEL7402 compared to Taxotere^® ^and non-targeted nanoparticles (DCT-nSLN). In vivo studies in hepatoma-bearing mice showed that the DCT-tSLN had a better therapeutic index compared to Taxotere^®^. Moreover, histological analysis demonstrated that DCT-tSLN had no detrimental effect on both healthy and fibrotic liver[129].

Human and murine macrophages express mannose receptor on their surface, [130] and several studies confirmed the feasibility of using mannose- or mannan-modified nanocarriers to target macrophages[131]. Alveolar macrophages play a key role in the first-line host defence and lung cell homeostasis, [132] thus targeting macrophages may provide innovative therapeutic strategies against tumour invasion and metastasis for lung cancer which represents one of the most aggressive solid cancers. Polysaccharides or multiple oligosaccharides, such as mannan, which contains a large group of mannose residues, are recognized as having a much higher affinity than single sugar molecules because of the moiety density[131]. Indeed, surface of DNA-loaded cationic SLN was modified with L-α-phosphatidylethanolamine (PE)-grafted mannan-based ligand (Mannan-PE) obtaining mannan-targeted SLN-DNA (Man-SLN-DNA)[131]. Transfection efficiency of Man-SLN-DNA was evaluated in vitro on RAW 264.7 cells (mouse leukemic monocyte macrophage cell line) and in vivo following pulmonary administration in rats. Man-SLN-DNA showed lower cytotoxicity than non-modified SLN-DNA and achieved higher gene expressions in comparison to Lipofectamine 2000-DNA. The above mentioned results indicated that mannan modification enhanced the active targeting ability of the carriers, and that Man-SLN-DNA may be a promising non-viral vector for targeted lung gene delivery[131].

Finally, taking advantages of overexpressed transferrin receptor at the surface of brain tumour cells, the surface of lipid nanocapsules has been coated with the OX26 murine monoclonal antibody and the NFL-TBS.40-63 peptide derived from the light neurofilament subunit (NFL)[133]. Intra-carotidal treatment with NFL-TBS.40-63 peptide functionalized nanocapsules was found to enhance the survival time (44 days versus 27 days) which was not obtained with non-targeted LNC. This suggests that this active targeting strategy may offer a promising approach for glioma treatment[133].

### Non-liposomal lipid-based nanocarriers for diagnostic (imaging) applications

The currently most accessible imaging techniques include magnetic resonance imaging (MRI), optical imaging, ultrasonography (US) and positron emission tomography (PET).

MRI is a powerful non-invasive technique based on magnetic properties, which offers the possibility of deep penetration into soft tissues. The human body consists by two-thirds of water molecules whose hydrogen atoms are able to act as microscopic compass needles susceptible to an externally applied magnetic field[134,135]. The different relaxation properties of various tissues allow using MRI to reconstruct images of structures, such as organs and lesions and to evaluate perfusion and flow-related abnormalities. MRI is optimized by using contrast agents able to increase the T_1 _signal or decrease the T_2 _signal, thus leading to a bright (positive) or dark (negative) contrast enhancement[136].

The electronic configuration (seven unpaired 4f electrons) of the lanthanide ion Gd^3+ ^allows to long electronic relaxation times or slower relaxation rates making it the most frequently T_1 _positive contrast agents for T_1_-weighted imaging in MRI[136]. The main drawback of Gd^3+ ^is its similarity with endogenous metals (e.g., calcium and zinc) that might cause transmetallation or neuromuscular transmission arrest[137]. In order to sequester the ion for a safe administration, cyclic (*e.g.*,cyclen-based tetraacetic acid derivative complex DOTA) or acyclic (*e.g.*,diethylenetriaminepentaacetic acid complexes DTPA) chelating agents have been approved for clinical use[136]. Lipid-based nanoparticulate carriers able to carry multiple contrast agent moieties were developed (*e.g.*, Gd-DTPA was encapsulate in SLN [138] or incorporated into the lipid layer of LDL-based nanoparticles [139])with the aim to increase the accumulation of the contrast agent at the target site, consequently enhancing the signal intensity,.

Contrast agents could also use of the ferromagnetic properties of natural elements (*i.e.*, iron), which consist in both being attracted in the presence of an externally applied magnetic field and retaining the magnetization after its removal. Superparamagnetic iron oxide nanoparticles (SPIOs) have been investigated as a category of T_2 _MRI contrast agents for both *in vitro *and *in vivo *imaging. They show a high magnetic moment that can increase proton relaxivities up to 10-folds[136].

Magnetic nanoparticles (MNPs)-encapsulated SPIOs have already demonstrated broader applicability and improved efficacy for the detection of primary tumours, metastasis, sentinel lymph node invasion and for the visualization of biological processes (e.g., apoptosis, cell trafficking, and gene expression)[21,140-142]. Biopharmaceutical performances, pharmacokinetics and toxicity depend on their composition and physicochemical properties as well as on the route of administration and dose (for review see [21]). Lipid-based nanocarriers have been suggested as a MRI contrast agent after encapsulation of MNPs[28,143-145].

Optical imaging is a non-invasive technique based on the specific optical properties of tissue constituents at different wavelengths[146]. The "biological window" for optical imaging in NIR region (wavelengths 700-900 nm) is characterized by low absorption and low scattering in soft tissue that allow increasing the penetration depth, the major limit in optical imaging[147].

Only two fluorophores (indocyanine green (ICG) and fluorescein) are currently approved by the FDA for medical use [148]. A successful optical molecular probe for medical imaging must show specific characteristics, such as absorption/emission wavelength in the deep or near infrared range, brightness, bio- and photo-stability and a successful pharmacokinetic profile (For review see [148]). Application of ICG is limited by its numerous disadvantageous properties, including its concentration-dependent aggregation, poor aqueous stability *in vitro*, low quantum yield and high binding to nonspecific plasma proteins, leading to rapid elimination from the body. To overcome these problems, ICG has been effectively encapsulated in lipid micellar systems, such as glycocholic acid and phosphatidylcholine [149] or phospholipid-PEG [149,150] micelles, improving ICG optical properties and prolonging up to a few weeks its stability in aqueous buffer. "Lipidots™", a recent technology based on oil-in-water nanoemulsions, in which a soybean oil and wax are coated with lecithin and PEG [151,152], has also been investigated to encapsulate near infrared dyes obtaining highly bright fluorescent nanoprobes with very low cytotoxicity and good pharmacokinetic profile *in vivo*[152,153]. For example, in a first clinical trial, ICG was successfully evaluated as a new method for sentinel lymph node biopsy in breast cancer patients [154] that represents an efficient aid to eradicate the tumour or prevent further metastasis[155].

In parallel, HDLs [55,156] and LDLs [107,157] have been modified by the inclusion of lipophilic fluorophores, such as DiR (1,1'-Dioctadecyl-3,3,3',3'-Tetramethylindotricarbocyanine Iodide), DiR-bis-oleate [55,156,157], carbocyanine-based optical probe (DiI) [107] or novel fluorescent lipids (such as bacteriochlorine_6_bisoleate (BchlBOA), a synthetic analog of Bacteriochlorophyll a (Bchl))[55,156]. Contrast generating materials can be included in the coating of the particle [157] or loaded in the hydrophobic core of lipoproteins[55,158].

Ultrasonography (US) is a low cost and in real time clinical imaging modality based on the partial backscattering of ultrasound waves - frequency range from 2 to 15 MHz - by different structures of the body because of the impedance mismatch between different tissues[159].

Due to the weak difference of echogenicity between different soft tissues, ultrasound contrast agents are usually needed to improve imaging and to distinguish between diseased and healthy tissues.

Perfluorocarbons (PFCs) are fluorinated aliphatic compounds that have been used as contrast agents for ultrasonography and magnetic resonance imaging (MRI) since the end of the 1970s[160]. Liquid PFCs (long perfluorinated carbon chain) have been used instead of gaseous PFCs (small perfluorinated carbon chain) due to higher resistance to pressure changes and mechanical stresses[161,162]. In order to administer liquid PFC by the parenteral route, nanoparticulate systems which encapsuled PFC droplets such as nanodroplets coated with phospholipid and cholesterol were designed [163,164].

Based on the use of a radiolabeled compound, Positron Emission Tomography (PET) is a non-invasive, nuclear imaging technique, capable of visualizing deep tissues with a high sensitivity and generating a three-dimensional image of living subjects. Using mathematical reconstruction methods and correction factors, quantitative information can be extracted from the images and radioisotope concentration can be measured in the specific region of interest[165].

Radiolabel SLNs with positron emitter ^64^Cu have been designed through the incorporation of a lipid-PEG-chelate (6-[p-(bromoacetamido) benzyl]-1,4,8,11-tetraazacyclotetradecane-N,N',N'',N'''-tetraacetic acid (BAT)), conjugated to a synthetic lipid, into the phospholipid monolayer forming the SLN surface[165]. The blood half-life of these SLNs was increased, comparatively to polymeric nanoparticles of similar size, due to reduced clearance in kidneys, liver and spleen.

Owing to the typical limitation for each technique, monomodal imaging was not enough for a successful diagnosis. In this context, multifunctional nanocarriers with plural imaging tools capabilities could be employed to exploit different modalities achieving molecular meaningful images at different levels of spatial resolution and dept. For example, functional multiplexed imaging with submicrometer resolution could be obtained using by optical imaging, although this technique does not provide quantitative concentration measurements and is basically restricted to biological objects no thicker than a few millimeters or centimeters. In contrast, PET allows quantitative whole body imaging with a low (a few mm) spatial resolution[166]. Taking advantage of multifunctional nanotechnology platforms, which include several contrast agents for multimodal imaging and tools for combining the different levels of observation, it was possible to reconcile molecular images into a global picture in order to the overcome the limit of each technique [28,166]. In this view, quantum dots nano-crystals (QDs) were encapsuled in functionalized phospholipid micellescovalently labeled with fluorine-18, a commonly used fluorophore for clinical imaging, developing a novel bifunctional probe for fluorescence and nuclear imaging [166]. Phospholipid QD micelles exhibited long circulation half-time in the bloodstream and slow uptake by the mononuclear phagocyte system, in contrast with several previous studies using other polymer coatings [166]. In addition, this bifunctional micellar probe showed that a combination of PET and fluorescence imaging can be used to quantitatively and dynamically improve the monitoring of nanoparticles biodistribution and pharmacokinetics. Despite that, toxicity due to the presence of heavy metals such as cadmium and selenium is their major concern. Unless QDs can be made especially small (around 6 nm) and thus excreted via the kidneys, these particles typically have delayed clearance and are mostly excreted through the liver and into the bile without significant metabolism[167].

Chen and co-workers have designed a novel multimodal tumour targeting molecular imaging probe encapsulating amphiphilic gadolinium chelates (Gd^3+^- Gadolinium diethylenetriaminepentaacetate-di (stearylamide)) and fluorescent dyes (DiR) in HDL-RGD targeted nanoparticle [168]. *In vitro *observation showed that specific HDL-RGD nanoparticles were preferentially taken up by endothelial cells escaping macrophage phagocytosis. RGD-targeted and untargeted HDL showed different accumulation/binding kinetics in mice-bearing subcutaneous human EW7 Ewing's sarcoma tumours. The combination of NIR and MR imaging exploits the complimentary features of both techniques providing high sensitivity and high spatial resolution[168].

Actively targeted contrast agent-loaded nanocarriers have also been developed to increase dye amounts at the tumour site. Since HDL's core lipid transfer is mediated through the interaction between ApoA-1, the major apolipoprotein, and the scavenger receptor class B type I (SR-BI), that is overexpressed in some cancer cell lines, [156,169] targeting this receptor represents a novel way to deliver imaging agents to tumours which overexpress this receptor. Furthermore, it is conceivable that a wide range of tumour-specific targets, such as epidermal growth factor (EGF), can be applied to HDL-like NPs[55,107,157]. A coordinated dual receptor (EGFR and SR-BI) targeting phenomenon leading to enhanced dye delivery has been shown by adding EGF targeting ligand to HDL-like NPs carrying DiR-BOA, a near-infrared fluorescent compound used as a model functional cargo[55].

Furthermore as mentioned above, LDL nanoparticles could reroute away from their native receptors by conjugating tumour-homing ligands to their surface. Proof of this strategy has been demonstrated *in vitro *with fluorescent-labeled folic acid-conjugated LDL[107]. Later, DiR-LDL-FA (actively targeted LDL obtained by intercalation of DiR into the LDL phospholipid monolayer and conjugation of FA to ApoB-100) have successfully targeted FR expressing tumours, thus effectively validating the LDL rerouting strategy for enhanced cancer optical imaging *in vivo*[157].

### Theranostics applications of non-liposomal lipid-based nanocarriers

**Figure 3:**
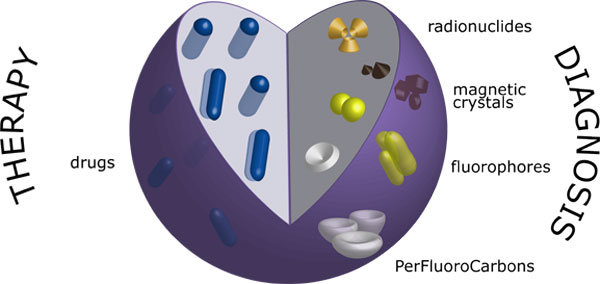
**Schematic representation of nanotheranostics**.

"Nanotheranostics" (i.e., theranostic nanomedicines) represent a novel extremely interesting versatile platform for both detection and cure of diseases, thanks to the development of multifunctional systems combing therapeutic and diagnostics functions(Figure 3)[16,170].

Taking advantage of the combination of simultaneous non-invasive diagnosis and treatment of diseases, one of the most promising aspects of the nanotheranostics is real time monitoring of pharmacokinetic drug profile to predict and validate the effectiveness of the therapy[18,171,172]. Due to these features, nanotheranostics are extremely attractive to optimize treatment outcomes in cancer, leading to the realization of a "personalized nanomedicine", which would enable to administer "the right drug to the right patient at the right moment"[173,174]. Significant benefits in the management of cancers could be achieved combining the highest therapeutic efficiency with the best safety profile[20].

HDL-like NPs for theranostic application were designed by incorporating a chemically stable bacteriochlorophyll analogue, a dye synthesized by the phototrophic bacteria, in their core [55,156]. This fluorescent photosensitizer can be tracked in vivo through NIR fluorescence imaging and can be activated to generate singlet oxygen upon light irradiation. NPs were successfully detected in epidermal carcinoma KB cells both in vitro and in tumour xenografts using the dorsal skinfold window chamber technique, which allows the monitoring of the nanoparticle tumour penetration with high spatial and temporal resolution [156]. Recently, Wang and co-workers proposed doxorubicin-loaded acoustic droplets containing a core of liquid perfluoropentane and lipid-based (DSPC, cholesterol, distearoylphosphatidylethanolamine (DSPE)-PEG2000) shell. High-intensity focused ultrasounds (HIFU) caused nanodroplets phase transition (i.e., acoustic droplet vaporization (ADV)) that led to the formation of gas bubbles, which mediated both mechanical cancer cell destruction and localized drug release, thus leading to significant cell toxicity[175]. Optical studies clearly illustrated the transient changes that occurred upon ADV of droplet-targeted and B-mode ultrasound imaging, revealing contrast enhancement by ADV in ultrasound images. Moreover, droplets were conjugated with aptamers, factitious oligonucleotides, providing the ability to specifically target CCRF-CEM human acute lymphoblastic leukaemia cells.

Gianella et al. [28] have developed a theranostic nanodevice composed of an oil in water nanoemulsion, loaded with iron oxide crystals, Cy7 dye and glucocorticoid prednisolone acetate valeranate, for MRI, NIRF and therapeutic use respectively. The effectiveness of this nanotheranostic, which combined the high spatial resolution of MRI with the high sensitivity of optical fluorescence imaging,was evaluated on a colon cancer model. The massive uptake of NPs in the tumour was confirmed in *in vivo* studies by MRI images, in which tumours appeared bright compared to the surrounding tissue, as well as by NIRF imaging since the injection of Cy7-labeled nanoemulsions led to a strong fluorescent signal compared to Cy7-unlabeled ones. RGD peptide-functionalized nanoemulsions resulted *in vivo *as active as the untargeted ones, due to the already extended tumour targeting of nanoparticles. Dayton and co-workers developed perfluorocarbon emulsion nanoparticles containing a core of at least 50% of liquid perfluorocarbons and a mixture of triacetin and soybean oils in which paclitaxel was encapsuled[29]. Another promising theranostic approach using ultrasound as imaging modality is represented by an oil-in-water emulsion made of liquid perfluoroctylbromide (PFOB) drops stabilized by a lipid layer in which the peptide melittin has been incorporated[176]. Melittin has already been proposed in the treatment of several cancers as cytotoxic agent that induces cell lysis through membrane permeabilization[177,178]. Feasibility as theranostic tool was investigated in vivo on xenograft models of breast cancer. Compared with control saline solution or mellitin free emulsion, the NPs treatment showed a significant inhibition of tumour growth. At the same time, they also provided a significant contrast enhancement, which enabled to monitor the therapeutic efficacy by ultrasound imaging[28,29].

Recently, Couvreur and co-workers reported a novel nanotheranostic platform in which SPIOs are coated with squalene-based anticancer prodrugs [144,145]. Lipid-drug conjugates have gained considerable attention in recent years thanks to the improvement of the pharmacokinetic and of the therapeutic index of the associated drugs. Squalene (SQ), which is a natural acyclic triterpene, is the corner stone in the biosynthesis of most triterpenes including lanosterol and cycloartenol which in turn are the precursors of steroids [179]. In 2006 the covalent linkage of the anticancer drug gemcitabine to squalene was found to lead to the formation of amphiphilic bioconjugates which spontaneously self-assembled as nanoparticles in water [180]. This proof of concept has since been enlarged to other nucleoside analogue drugs (e.g., ddI, ddC, AZT, ACV, Ara-C [180-184]), to more lipophilic drugs (e.g., paclitaxel [185,186]), to imaging transition metals (e.g., ruthenium[187] or gadolinium[188]) as well as to antibiotics (e.g., penicillin [61])and nucleic acids (e.g., SiRNA[189]).

**Figure 4:**
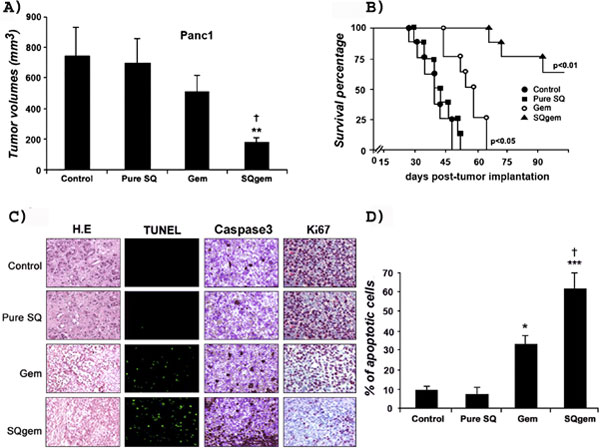
***In vivo* antitumor efficacy of SQgemcitabine NP**. (A) Mice bearing pancreatic chemoresistent Panc1 orthotopic tumour model were treated with equivalent drug dose of gemcitabine (dFdC) or SQgemcitabine (SQdFdC). After 1 month of treatment, volume of the primary tumour and tumour extension were significantly reduced by SQgemcitabine showing its superior antitumour efficacy compared to physiological solution or vehicle nanoparticles treated (pure SQ) or gemcitabine treated mice. (B) Mice survival curves showed a significant enhancement of the median survival after SQgemcitabine treatment. All the gemcitabine treated and untreated mice died respectively within 64 and 47 days following tumour implantation. Remarkably, mice treated with SQgemcitabine were still alive after 3 months and no tumours were detected after autopsy. (C) Tumour biopsy samples were collected from each group of mice and used for immunohistochemistry examination. Paraffin sections submitted to hematoxylin-eosin (H.E) from SQgemcitabine treated mice revealed an absence of mitotic figures and demonstrated enlarged cells with significant necrotic changes. Tissues staining with terminal deoxynucleotidyltransferase (TUNEL), for detecting DNA fragmentation, and aspartic acid-specific cysteine proteases (CASPASE3), that are both present during apoptotic signaling cascades, revealed that apoptosis was most prominent in animals treated with SQgemcitabine. The number of Ki-67-positive tumour cells, a marker for proliferation, showed a significant decrease of the tumour proliferative activity in SQgemcitabine in comparison to gemcitabine treatment. (D) Quantitation rates of apoptotic cells confirmed the considerably increased apoptosis in the tumours from SQgemcitabine-treated mice and the statistically significant difference between SQgemcitabine and gemcitabine treatment. Adapted from ref 176. Copyright 2011 Nanomedicine.

In general, these squalenoylated nanomedicines displayed an increased pharmacological activity in solid, metastatic and orthotopic experimental cancers (Figure 4)[190]. When the SPIOs/SQgemcitabine NPs were intravenously injected in the tumour-bearing mice and guided using an extracorporeal magnetic field, an impressive anticancer activity was obtained at very low doses of the anticancer drug. Moreover, the magnetic responsiveness of embedded SPIOs coupled to their T2 imaging properties make them an efficient candidate for theranostic applications, because tumour collapse could be easily visualized by MRI[145]. This concept has also been found feasible by using Gd3+ for T1 positive imaging[188], showing that the squalenoylation is a versatile and safe nanotheranostic platform with high drug loading and controlled release properties.

## Conclusions

Lipids are a class of natural or synthetic compounds with a range of structure and functions. Their supramolecular organization may be tailored to design nanoscaled structures able to be loaded with drugs or imaging agents or both ("nanotheranostics"). The proof of concept that such lipid nanocarriers may be used for cancer treatment and diagnosis is demonstrated in the present review.

## Competing interests

The authors declare that they have no competing interests.
